# Nebulized step-down budesonide vs. fluticasone in infantile asthma: A retrospective cohort study

**DOI:** 10.3892/etm.2020.9140

**Published:** 2020-08-25

**Authors:** Zhimin Wu, Xiangli Bian, Lei Hui, Jinping Zhang

Exp Ther Med 19:1665–1672, 2020; DOI: 10.3892/etm.2019.8401

After the publication of the above paper, the authors have realized that the affiliation was presented incorrectly in their paper. The affiliation should have been presented as follows (changes are highlighted in bold): “Department of Pediatrics, **Shanghai Sixth People’s Hospital East Affiliated to Shanghai University of Medicine & Health Sciences**, Shanghai **201306**, P.R. China”. The authors regret that this error with the author affiliations was not noticed prior to the publication of their paper, and apologize for any inconvenience caused.

